# Association of Ultra-Processed Food Intake with Insulin Secretion and Sensitivity in Mexican American Children: Results from the SAFARI Study

**DOI:** 10.3390/nu18142361

**Published:** 2026-07-18

**Authors:** Hemant Kulkarni, Rector Arya, Srinivas Mummidi, Sharon P. Fowler, Roy G. Resendez, Juan Carlos Lopez-Alvarenga, Alvaro Diaz-Badillo, Jennifer Schneider, Sobha Puppala, Vidya Farook, Daniel E. Hale, Jane L. Lynch, Bradley C. Johnston, Vijay Golla, Donna M. Lehman, Ralph A. DeFronzo, John Blangero, Ravindranath Duggirala

**Affiliations:** 1 Department of Health and Behavioral Sciences, College of Arts and Sciences, Texas A&M University-San Antonio, One University Way, San Antonio, TX 78224, USA; rarya@tamusa.edu (R.A.); smummidi@tamusa.edu (S.M.); rresendez@tamusa.edu (R.G.R.); abadillo@tamusa.edu (A.D.-B.); jschneider@tamusa.edu (J.S.); dlehman@tamusa.edu (D.M.L.); rduggirala@tamusa.edu (R.D.); 2 Department of Medicine, Joe R. & Teresa Lozano Long School of Medicine, University of Texas Health San Antonio, 7703 Floyd Curl Drive, San Antonio, TX 78229, USA; fowlers@uthscsa.edu; 3 Division of Population Health and Biostatistics, Department of Primary & Community Care, School of Medicine, University of Texas Rio Grande Valley, 2102 Treasure Hills Blvd, Harlingen, TX 78550, USA; juan.lopezalvarenga@utrgv.edu; 4 Center for Precision Medicine, Wake Forest University School of Medicine, Winston-Salem, NC 27157, USA; sobha.puppala@advocatehealth.org; 5 Department of Human Genetics and South Texas Diabetes and Obesity Institute, College of Sciences, University of Texas Rio Grande Valley, Office 2.233, Biomedical Research and Health Building, One W University Boulevard, Brownsville, TX 78520, USAjohn.blangero@utrgv.edu (J.B.); 6 Department of Pediatrics, Penn State Health and Penn State College of Medicine, Penn State University, 35 Hope Dr., Hershey, PA 17033, USA; dhale2@pennstatehealth.psu.edu; 7 Department of Pediatrics, Joe R. & Teresa Lozano Long School of Medicine, University of Texas Health San Antonio, 7703 Floyd Curl Drive, San Antonio, TX 78229, USA; lynchj2@uthscsa.edu; 8 Department of Nutrition, College of Agriculture and Life Science, Texas A&M University, College Station, TX 77840, USA; bradley.johnston@exchange.tamu.edu; 9 Department of Environmental and Occupational Health, School of Public Health, Texas A&M University, 212 Adriance Lab Rd, College Station, TX 77843, USA; vijay.golla@tamu.edu; 10 Division of Diabetes, Department of Medicine, Joe R. & Teresa Lozano Long School of Medicine, University of Texas Health San Antonio, 7703 Floyd Curl Drive, San Antonio, TX 78229, USA; defronzo@uthscsa.edu

**Keywords:** ultra-processed food, diabetes mellitus, insulin secretion, insulin sensitivity, insulin resistance, family studies, children

## Abstract

**Background/Objectives:** There is increasing interest in and evidence for the role of ultra-processed foods (UPFs) in cardiometabolic risk. We tested the hypothesis that UPF intake score derived from dietary history is associated with measures of insulin secretion and sensitivity in a family setting even when the process of type 2 diabetes is still to ensue. **Methods**: We used data from our well-characterized San Antonio Family Assessment of Metabolic Risk Indicators in Youth (SAFARI) study cohort of nondiabetic children (aged 6–17 years). Dietary history was assessed using the Block Food Frequency Questionnaire. Outcomes of interest were 19 clinically useful indexes of insulin secretion/sensitivity based on fasting and oral glucose tolerance test results. Since the SAFARI study employed a family study design, we used polygenic regression models that adjusted for complex interactions between age and sex and accounted for important comorbidities. **Results**: A total of 53.3% of daily energy intake was contributed by UPF. Of the 19 indexes tested for association with the UPF score, we found a statistically significant association with measures of insulin resistance and beta cell function and with three indexes based on the results of oral glucose tolerance test as follows: Matsuda index (β = −0.0962, *p* = 0.0262), insulinogenic index at 30 min (β = 0.1779, *p* = 0.0006) and disposition index at 30 min (β = 0.1538, *p* = 0.0061). **Conclusions**: Even in nondiabetic children there was a significant and independent association of UPF intake with insulin secretion and sensitivity. Future studies need to investigate this association in larger, longitudinal settings and in randomized trials controlling potential systematic errors.

## 1. Introduction

The past decade has seen a worldwide sudden increase in ultra-processed food (UPF) intake, with particularly high intake reported in countries such as the United States and the United Kingdom, although important variations exist according to age, sex, and body mass index (BMI) [1]. The UPF market has also expanded in the middle- and low-income countries, driven in part by urbanization and policies that facilitate foreign investment in the food industry [2]. In the United States, UPF consumption has continued to increase across successive NHANES cycles, with estimates suggesting an approximately 1% increase per survey cycle [3]. Children and adolescents derive a substantial proportion of their daily caloric intake from UPFs, making this age group particularly relevant for studying the early metabolic consequences of dietary exposures [4].

Several systems have been proposed to classify processed foods, but the NOVA classification remains the most widely used. NOVA categorizes foods into four groups according to the extent and purpose of industrial processing. Group 4 UPF includes formulations manufactured largely from industrial ingredients and additives that are rarely used in home cooking, such as hydrolyzed proteins, high-fructose corn syrup, maltodextrin, hydrogenated oils, flavor enhancers, and cosmetic additives [5].

The increase in UPF consumption has been shown to be associated with a concomitant rise in the risks associated with cardiometabolic health [6,7,8,9]. This influence is multifactorial—UPFs tend to have high carbohydrate content, reduced fiber content, hyper-palatability, rapid digestibility, and are associated with alterations in gut microbiota composition [10], increased intestinal permeability, and low-grade chronic inflammation [11,12]. These pathways may contribute to metabolic stress and impair the physiological regulation of glucose homeostasis [13]. In addition, the use of UPF may influence insulin action through repeated exposure to rapidly absorbable carbohydrates, lower satiety [14], alterations in incretin signaling, and chronic inflammation. Together, these component mechanisms elevate metabolic stress and modify a normal pancreatic response to food intake.

Most epidemiologic studies on ultra-processed food intake have focused on obesity, metabolic syndrome or type 2 diabetes as clinical outcomes. These conditions represent late manifestation of metabolic problems. Alterations in insulin sensitivity and compensatory insulin secretion appear years before the onset of hyperglycemia [15,16] and can be quantified using dynamic indexes derived from oral glucose tolerance tests, thereby providing a sensitive window to catch early metabolic effects of dietary exposures. The dynamic indexes obtained from OGTT capture complementary aspects of insulin sensitivity and beta-cell function [17]. Moreover, the analysis in a family-based cohort offers the opportunity to evaluate metabolic phenotypes, considering shared genetic and environmental influences that may confound dietary associations.

Since early childhood is a critical period for metabolic development, the effects of UPFs on insulin and glucose regulation may be detectable before disease develops. We hypothesized that higher UPF consumption would be associated with reduced insulin sensitivity and compensatory alterations in β-cell function, detectable through OGTT-derived indexes, even among children without diabetes. To test this hypothesis, we analyzed data from non-diabetic children participants enrolled in the San Antonio Family Assessment of Metabolic Risk Indicators in Youth (SAFARI) Study [18].

## 2. Materials and Methods

### 2.1. Study Participants

The present study is a secondary data analysis of the original SAFARI study [18]. The inclusion criteria for study participants in the SAFARI study were: children and teenagers aged 6 to 17 years old whose adult members had taken part in one of three family-based genetic epidemiology studies—the San Antonio Family Heart Study (SAFHS), San Antonio Family Diabetes/Gallbladder Study (SAFDGS), or the Veterans Administration Genetic Epidemiology Study (VAGES). The participants of these parental studies represented predominantly lower-income Mexican American families in San Antonio (Mitchell et al. 1996 [19]; Puppala et al. 2006 [20]; Coletta et al. 2009 [21]; Fowler et al. 2013 [18]). Enrollment of study participants took place between September 2005 and May 2010 [18]. The original SAFARI study was conducted to address the burden of CO and its associated cardiometabolic risk in children and adolescents. Briefly, a total of 673 children and adolescents (SAFHS = 373, SAFDGS = 126, VAGES = 174) participated in the SAFARI study, representing 401 nuclear families/sibships. Each sibship contained an average of approximately 2 (range: 1–5) children. These sibships were embedded within the original SAFHS, SAFDGS, and VAGES extended families, representing the youngest of multiple generations from their families to have taken part in the three parental studies [18]. Disturbingly, SAFARI children were found at high-risk of health outcomes (obesity 34%, metabolic syndrome 19%, and pre-diabetes 13%) [18]. It should be noted that the original SAFARI study enrolled participants in a family-based, proband-driven ascertainment protocol.

For the present analyses we selected subsets of data where information on dietary assessment and measures of insulin secretion and sensitivity were available—participants with missing data on these key variables were not included in the analysis. As the SAFARI study was conducted in pediatric age group, written informed consent from both parents was obtained and children aged 7 years and above also provided signed assent prior to initiation of assessment. This study was conducted following the principles of Declaration of Helsinki and approved by the Institutional Review Board (or Ethics Committee) of University of Texas Health San Antonio (CR00000754 and 8/15/2025).

### 2.2. Dietary Assessment

Children enrolled in the SAFARI study also underwent dietary assessment using the well-characterized and validated Block Kids’ Food Frequency Questionnaire (FFQ) [22,23]. These data were available for 508 SAFARI study participants. The resulting information from 78 questions regarding the consumption of food/beverage items (frequency and amounts consumed) was analyzed by NutritionQuest (Berkeley, CA, USA). UPFs were defined as in the Nova classification [24]. Nova classification categorizes foods into the following four categories—unprocessed or minimally processed (Nova 1), processed culinary ingredients (Nova 2), processed foods (Nova 3) and ultra-processed foods (Nova 4). For this study, a food item was considered ultra-processed if any one of the following two criteria were met: an Environmental Working Group (https://www.ewg.org/, accessed on 1 November 2025) food score of 4 or more or presence of at least one Nova 4 ingredient. UPF intake was semi-quantitatively estimated by calculating a UPF score which used the following formula: $\sum_{u=1}^{U}f_{u}a_{u}$, where *f* indicates the frequency and *a* the amount ingested in each serving over the past week and *U* represents the subset of UPFs from a list of 61 food items included in the Block FFQ. All analyses presented here use the raw UPF score without adjustment for calorie intake.

### 2.3. Measures of Insulin Secretion and Sensitivity

Based on the clinical evaluation of enrolled participants, we computed a total of 19 indexes of insulin sensitivity and secretion as outcome measures, the basis and calculations of which are detailed by Hudak et al. [25]. Of the 19 included indexes, nine were measured during fasting (fasting glucose, fasting insulin, QUICKI, HOMA_β, HOMA_s, HOMA_IR, TyG index, serum C peptide and impaired fasting glucose) and ten were estimated from results of oral glucose tolerance tests (OGTT, area under the curve (AUC) for glucose, AUC for insulin, Matsuda insulin sensitivity index [MatISI], insulinogenic index 30, disposition index 30, insulinogenic index 120, disposition index 120, ISI_0,120_, Bogalusa insulin sensitivity index and impaired glucose tolerance). All study procedures have been approved by the Institutional Review Board of the University of Texas Health San Antonio, San Antonio, Texas, as mentioned above.

### 2.4. Statistical Analysis

Since the SAFARI study used a family-based design, we used a variance component method that partitions variance of a trait into genetic, associative and environmental components. Also, in the absence of knowledge about specific gene-based associations, the influence of genetics on a selected trait is assumed to originate from polygenes. A commonly used regression model that permits inclusion of these effects is the polygenic model. Hypotheses for all associations were tested using polygenic models of the form—$O_{i}=m+\sum b_{k}a_{ik}+g_{i}+e_{i}$—where *O* is the outcome of interest; *m* is the trait mean; *a* is the covariate vector of dimension *k* with *b* as the corresponding regression coefficients; *g* is the polygenic effect (used to estimate heritability, h^2^r) and *e* is the residual error for an individual indexed by *i* [26]. The proportion of the genetic (g) to the total trait variance refers to heritability which, in a broad sense, quantifies the contribution of polygenes to trait variability. In all models we included age, age^2^, age*sex, age^2^*sex, sex, body mass index, waist circumference, systolic and diastolic blood pressure, total serum cholesterol, high-density lipoprotein (HDL) cholesterol, serum triglycerides and physical activity (MET) scores as covariates. Further, pubertal status of the children can exert a confounding effect on some phenotypic associations in the SAFARI study [18]. Therefore, we included pubertal status as a covariate in all models.

We considered the possibility that the association of UPF intake with indicators of insulin secretion and sensitivity may, in part, be either confounded or mediated by the total calorie intake. This possibility is important to consider since our UPF score was not adjusted for calorie intake. Due to the exploratory nature of this study, we did not conduct formal mediation analyses; however, we conducted an additional set of sensitivity analyses by including total calorie intake as a covariate in the polygenic regression models and thus adjusting for calorie intake through regression modeling.

All the traits—outcome as well as predictors—were inverse-normalized to ensure model requirements of parametric nature. Association with dichotomous traits (impaired fasting glucose and impaired glucose tolerance) was assessed using a liability threshold modeling approach. Descriptive statistics were generated using Stata 19.0 (Stata Corp, College Station, TX, USA) and association analyses were conducted using the Sequential Oligogenic Linkage Analysis Routines (SOLAR)-Eclipse software package (version 8.5.1, http://www.nitrc.org/projects/se_linux, accessed on 10 October 2025) and dedicated Tcl scripts.

## 3. Results

### 3.1. Characteristics of the Study Participants

Table 1 provides a detailed summary of characteristics of the study participants. Briefly, the average age of study participants was 11.5 years, 49% were females and average BMI was 22.67 kg/m^2^. The prevalences of general and abdominal obesity exceeded 30% each while that of glucose intolerance (i.e., IFG: impaired fasting glucose, IGT: impaired glucose tolerance; pre-diabetes: IFG, IGT or both), high blood pressure, dyslipidemia and metabolic syndrome was 13%, 12%, 32%, and 19%, respectively.

Pairwise kinship pattern showed that out of a total 3664 kinship pairs the most common were third cousins (662 pairs), second cousins (661 pairs), first cousins (550 pairs), and siblings (383 pairs) [18]. As shown in Table 1, male and female participants significantly differed from each other with respect to systolic blood pressure, physical activity score, fasting glucose concentration, HOMA_β, and OGTT-based insulin indices such as Matsuda index, insulinogenic index and disposition index.

### 3.2. Heritability of Insulin-Related Traits

Of interest, the heritability of all the 19 traits was high to very high, suggesting a dominant genetic component in the variability of insulin secretion and sensitivity (Table 2). For example, except for insulinogenic index at 120 min and the Matsuda insulin sensitivity index (MatISI), all other indexes measured on a continuous scale reached statistical significance with estimated heritability exceeding 0.43. On the other hand, polygenic models for serum C peptide and the ISI_0,120_ indexes could not reliably estimate heritability. These results also provided the rationale for using polygenic regression models since they adjust the association regression coefficients for the observed genetic influence on traits studied.

### 3.3. Association of UPF Scores with Outcomes

**Figure 1:**
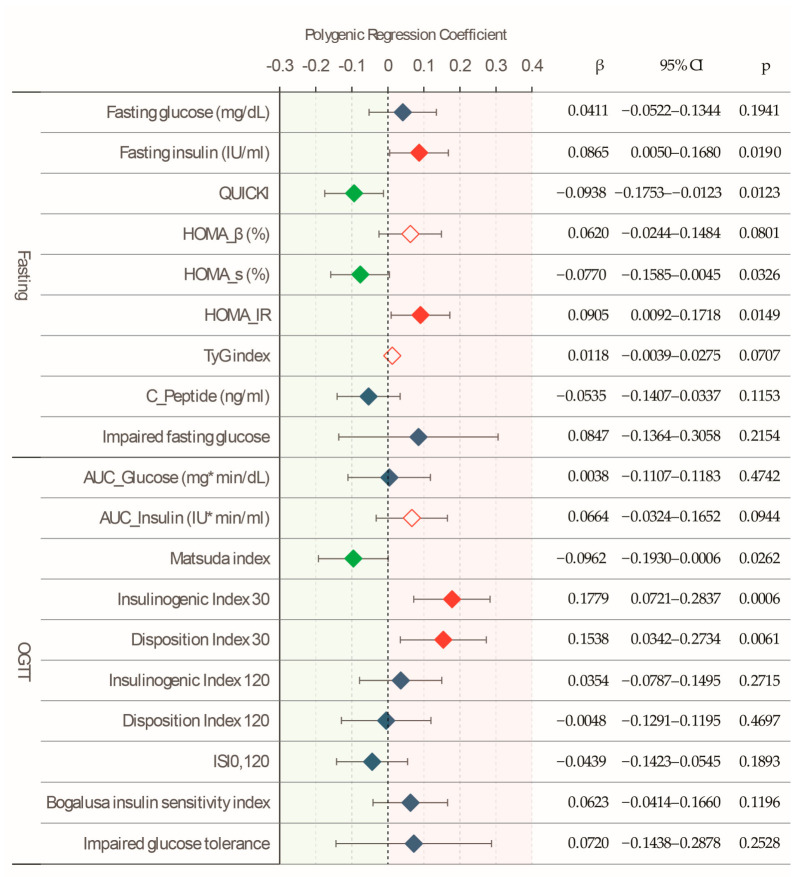
Forest plot showing polygenic regression coefficients for the association of UPF score with indices of insulin secretion and sensitivity. Diamonds represent point estimates and error bars the 95% confidence intervals. Solid green diamonds indicate significantly negative coefficient, solid red diamonds indicate significantly positive association, solid blue diamonds represent non-significant regression coefficients, and hollow red diamonds indicate regression coefficients with *p*-values between 0.05 and 0.10. Light pink and green background colors represent a positive and negative association. All models are adjusted for age, age^2^, sex, age*sex, age^2^*sex, body mass index, waist circumference, systolic and diastolic blood pressure, total serum cholesterol, HDL cholesterol, serum triglycerides, metabolic equivalent (MET) scores for physical activity and pubertal status. β, polygenic regression coefficient; SE, standard error; *p*, significance value.

We observed that 53.3% of the energy intake of the study participants came from UPF. Association analyses indicated that after adjusting for pedigree structure, first and second order interactions between age and sex and indicators of metabolic conditions, level of physical activity and pubertal status, a high UPF score was significantly associated with seven of the 19 traits (Figure 1).

Specifically, in the context of indices measured during fasting, the UPF score was significantly negatively associated with insulin sensitivity as measured by HOMA_s (β = −0.077, *p* = 0.0326) as well as QUICKI (β = −0.0938, *p* = 0.0123) and positively associated with fasting insulin (β = 0.0865, *p* = 0.0190) and insulin resistance as measured by HOMA_IR (β = 0.0905, *p* = 0.0149). With respect to OGTT-based indices, the UPF score was significantly inversely associated with the Matsuda index (β = −0.0962, *p* = 0.0262) and positively with insulinogenic index 30 (β = 0.1779, *p* = 0.0006) and disposition index 30 (β = 0.1538, *p* = 0.0061). In addition, the UPF score was associated with two other indices of insulin secretion (HOMA_β and AUC_Insulin) and one index of insulin resistance (TyG) with *p*-values between 0.05 and 0.1. Together, these results show that even during childhood in nondiabetic children, a measure of UPF consumption was clearly associated with a lower insulin sensitivity, a higher insulin secretion and higher insulin resistance.

### 3.4. Sensitivity Analyses

When we included total dietary calorie intake as a covariate in all the models, we observed (Supplementary Table S1) an interesting change in the pattern of association of UPF intake with measures of insulin secretion and sensitivity. We found that all the fasting indexes that used the information of serum insulin concentration (fasting insulin, all HOMA measures, and QUICKI) showed a weakened, non-significant association with UPF intake (compare results from Figure 1 and Supplementary Table S1). On the other hand, fasting indexes that used glucose but not insulin (fasting glucose, TyG and IFG) showed stronger and significant association with UPF intake. In the context of the OGTT-related indexes, the Mitsuda index lost its statistical significance after adjusting for total calorie index (Supplementary Table S1) but the insulinogenic index and disposition index at 30 min retained their statistical significance (*p* = 0.0091 and 0.0085, respectively).

## 4. Discussion

Food processing is designed to improve the quality, digestibility and shelf-life of food. However, since food processing is, by definition, artificial in nature, it poses metabolic challenges. For example, UPFs tend to contain less water, more preservatives and food dyes [5]. Our results using dietary questionnaire data aimed at exploring food intake patterns showed that even in nondiabetic children, ingestion of UPFs was associated with altered indexes of insulin secretion and sensitivity. Specifically, during OGTT we observed that MatISI, insulinogenic index and disposition index (measured 30 min after glucose stimulus) were adversely influenced by UPF intake. It should be noted, however, that since a total of 19 traits were assessed in this study, if one were to apply the rigid Bonferroni correction for multiple comparisons, then an allowable type I error rate would be 0.0026. Using that strict threshold would indicate that UPF intake was significantly associated with indexes of a 30 min, post-load pancreatic response. Thus, our results indicate that UPF intake is likely detrimental to insulin secretion and sensitivity.

These results agree with the increasing body of the literature supporting the detrimental influence of UPFs on children. Vallianou et al. [27,28]. provide an elegant review of human studies and a detailed description of the multiple ways in which UPF intake can influence metabolic pathophysiology. These include higher content of easily digestible carbohydrates and fats, addition of emulsifiers such as carrageenan, contamination with byproducts like acrylamide and bisphenol-A and storage leaks of plasticizers like phthalates. Moreover, Edalati et al. [29]. have shown that UPF intake can potentially be associated with DNA damage. Interestingly, reviews by both Santos et al. [30]. and Miranda et al. [31]. surmise that while there is a burgeoning body of the literature supporting an association between UPF intake and metabolic conditions, not all studies show it consistently. In an additional set of nine studies that we reviewed, six studies reported associations between UPF intake and increased problems with glucose homeostasis, specifically, while all nine studies reported associations between UPF intake and one or more measures of increased cardiometabolic risk.

For example, the study by Li et al. [32]. conducted in Southern California, USA, reported that 10% increase in UPF intake in 17–22 year olds was associated with a 51% increase in risk of prediabetes and 158% increase in the risk of glucose intolerance. Three studies from Brazil have demonstrated that a reduction in UPF intake reduced glucose, insulin and HOMA-IR independent of the influence on BMI [33], and in preschool children there was a strong association with measures of adiposity [34] and dyslipidemia [35]. Similarly, studies from China [36], Iran [37], Chile [38], Republic of Korea [39] and Spain [40] have consistently shown the detrimental influence of UPF intake on the metabolic health of children.

Very little is known about the mechanisms by which UPF intake can influence insulin biology in humans. Our study has two strengths—first, using a family study design, our study accounts for the genetic component of insulin secretion and sensitivity. Second, using a battery of insulin-related indexes permits us to seek biologically meaningful associations of UPF intake with insulin dysfunction or dysregulation. As shown in Table 1, there was an indicative association of the UPF score with fasting insulin levels as well as measures of insulin resistance (HOMA_IR) and sensitivity (QUICKI, HOMA_s). On the other hand, a stronger and significant association was observed with post-glucose load estimation of insulin sensitivity (MatISI) and secretion (insulinogenic index and disposition index). To our knowledge, such a pattern of association has not been previously described.

The change in patterns of association after adjusting for calorie intake in the polygenic regression models raises interesting hypotheses for future research. Our results indicated that from a mechanistic standpoint, UPF intake may be associated with insulin secretion and sensitivity indirectly through the association of calorie intake. On the other hand, the fact that three indexes that used fasting glucose (fasting glucose, TyG and IFG) without fasting insulin showed an improved and stronger association with UPF intake thereby raising the hypothesis that UPF intake may be associated with fasting glucose levels via calorie-independent mechanisms. Previous studies support both these hypotheses [41,42,43]. Still, the fact that insulinogenic index and disposition index retained their statistical significance even after adjusting for total calorie intake indicates that the direct influence of UPF intake on insulin secretion cannot be refuted.

Our study has limitations. Firstly, our study represents a cross-sectional estimate of UPF intake and insulin function and therefore causative interpretation cannot be drawn. Secondly, the SAFARI study was not primarily designed to measure or quantify UPF intake, but these data were derived from the Block Food Frequency Questionnaire. Although the Block Kids’ FFQ was not originally designed to classify foods by UPF content, it has been successfully used previously to derive and quantify UPF content [44]. Despite this, we acknowledge the likelihood of a potential misclassification with respect to ultra-processed food intake. An additional source of potential misclassification could have originated in our study by using EWG scores. As explained on their website (https://www.ewg.org/foodscores/content/methodology/, accessed on 9 July 2026) the EWG scores were generated to classify healthful versus less healthful foods based primarily on nutritive content, presence of contaminants and degree of processing. As a result, the EWG score could have introduced a bias in favor of UPF intake. Third, UPFs represent a conglomerate variety of foods, and it is now recognized that the hyperpalatable subset of UPFs is more likely to influence metabolic outcomes than other UPFs. Our study could not estimate the hyper-palatability of UPFs. Fourth, our data come from non-diabetic children. Therefore, these results cannot be directly generalized to a diabetic population. However, since these children were at a high risk of future metabolic risks the results of the study provide mechanistic insights. Fifth, the strengths of associations were estimated using available data often with different sample sizes for the traits studied. Therefore, comparison of strengths of association across the 19 traits cannot be done. Sixth, the SAFARI study and the dietary assessments were conducted just over 20 years ago. The methods of food processing have substantially changed over the past 20 years. Therefore, the findings from this study should only be considered as a proof-of-principle and not a generalization applicable to current methods of food processing. Seventh, we studied multiple associations (UPF intake with 19 traits). Since the primary nature of this study was to exploit available data to explore putative associations, we did not account for multiple testing correction. All our findings should thus be considered indicative but not confirmatory. Eighth, there was a substantial reduction in sample size given the analyses for OGTT-based tests. We therefore compared key characteristics of study participants who were able to complete the OGTT to the entire group of study participants and found (Supplementary Table S2) that there was no evidence for a biased departure for any characteristic due to the reduced sample size for OGTT-based indexes. Ninth, the observed pattern of association between UPF score and measures of insulin sensitivity and secretion is—implicit in the polygenic models used—independent of the strong genetic influence on the insulin-related traits. However, the statistical possibility of a gene x environment interaction cannot be excluded. Indeed, future studies need to tease out the gene x environment (UPF score) interactions and their putative influence on insulin biology.

## 5. Conclusions

Notwithstanding the limitations, our study raises the hypothesis that UPF intake may have a deleterious influence on both insulin secretion and sensitivity that is independent of a potential genetic concurrence or existing comorbidities. Our observations support our study hypothesis that the influence of UPF intake on insulin secretion and sensitivity may be detectable early in life. Future studies need to delineate these mechanistic insights in larger, longitudinal contexts and we plan to recall the SAFARI study participants in the near future to assess our findings in a longitudinal framework.

## Figures and Tables

**Table 1:** Characteristics of the study participants (N = 508) on whom dietary information was available.

Characteristic	Males		Females		*p*
	N	Mean (SD)/N (%) *	N	Mean (SD)/N (%)	
Age (y)	260	11.40 (3.44)	248	11.41 (3.51)	0.9852
BMI (kg/m^2^)	259	22.31 (6.08)	248	22.30 (6.24)	0.9946
Waist circumference (cm)	260	75.91 (18.0)	244	75.48 (17.4)	0.7844
Systolic blood pressure (mmHg)	260	105.55 (10.42)	248	102.36 (9.33)	0.0003
Diastolic blood pressure (mmHg)	260	62.49 (7.26)	248	63.05 (7.07)	0.3841
Total serum cholesterol (mg/dL)	246	148.24 (27.77)	228	147.17 (25.88)	0.6655
Serum high density lipoprotein cholesterol (mg/dL)	245	46.05 (11.44)	227	44.67 (10.31)	0.1720
Serum triglycerides	242	72.15 (35.80)	227	75.63 (35.63)	0.2927
Physical activity (MET score)	247	2.24 (1.33)	230	1.85 (1.24)	0.0009
UPF score	260	119.97 (33.68)	248	121.22 (33.56)	0.6749
Fasting glucose (mg/dL)	247	91.01 (7.33)	233	88.39 (6.56)	<0.0001
Fasting insulin (IU/mL)	244	12.30 (6.78)	228	13.35 (7.19)	0.1014
QUICKI	242	0.34 (0.03)	227	0.33 (0.03)	0.4767
HOMA_β (%)	246	139.99 (57.10)	229	156.21 (63.22)	0.0035
HOMA_s (%)	246	71.68 (45.13)	229	68.15 (45.88)	0.3984
HOMA_IR	244	1.80 (0.96)	227	1.93 (0.99)	0.1732
TyG index	238	7.99 (0.48)	226	8.01 (0.46)	0.6580
C_Peptide (ng/mL)	209	0.93 (0.80)	194	0.98 (0.71)	0.4691
Impaired fasting glucose	249	19 (7.63)	233	10 (4.29)	0.1235
AUC_Glucose (mg*min/dL)	155	8.25 (1.13)	148	8.10 (1.11)	0.2238
AUC_Insulin (IU*min/mL)	149	6.41 (6.32)	140	7.51 (6.23)	0.1399
MatISI	145	3.88 (1.62)	138	3.42 (1.38)	0.0113
Insulinogenic index 30	149	1.68 (1.17)	140	2.25 (1.72)	0.0010
Disposition index 30	146	5.65 (3.45)	137	6.88 (4.96)	0.0157
Insulinogenic index 120	155	1.48 (6.48)	147	4.46 (14.40)	0.0161
Disposition index 120	147	4.61 (25.72)	138	13.57 (49.32)	0.0534
ISI_0,120_	151	87.61 (28.99)	144	82.39 (30.22)	0.1316
Bogalusa insulin sensitivity index	156	504.44 (684.10)	144	591.37 (724.53)	0.2860
Impaired glucose tolerance	157	20 (12.74)	150	18 (12.00)	0.8442

*, cells show mean (SD) for continuous variables and N (%) for categorical variables.

**Table 2:** Heritability of the insulin secretion and sensitivity indices estimated using polygenic modeling in the SAFARI study. All models are adjusted for age, age^2^, sex, age*sex, age^2^*sex, body mass index, waist circumference, systolic and diastolic blood pressure, total serum cholesterol, HDL cholesterol, serum triglycerides, metabolic equivalent (MET) scores for physical activity and pubertal status.

Trait Group	Trait *	Mean	SD	N	Heritability			
					h^2^r	SE (h^2^r)	95% CI **	*p*
Fasting	Fasting glucose (mg/dL)	89.63	7.03	434	0.7148	0.1442	0.4322–0.9974	0.0000
	Fasting insulin (IU/mL)	12.99	7.23	430	0.6341	0.1513	0.3376–0.9306	0.0000
	QUICKI	0.34	0.03	428	0.5886	0.1505	0.2936–0.8836	0.0000
	HOMA_β (%)	150.40	63.62	434	0.5405	0.1511	0.2443–0.8367	0.0001
	HOMA_s (%)	69.43	45.56	434	0.4691	0.1526	0.1700–0.7682	0.0007
	HOMA_IR	1.89	1.01	430	0.6274	0.1506	0.3322–0.9226	0.0000
	TyG index	7.99	0.47	434	0.6921	0.1404	0.4169–0.9673	0.0000
	C_Peptide (ng/mL)	1.00	0.92	370	1.0000 #	0.0000	---	0.0000
	Impaired fasting glucose ^†^	0.06	0.24	436	0.9655	0.7178	0.0000–1.0000	0.1041
OGTT	AUC_Glucose (mg*min/dL) × 100	8.15	1.13	274	0.6100	0.2051	0.2080–1.0120	0.0016
	AUC_Insulin (IU*min/mL) × 1000	7.04	6.36	262	0.9294	0.2285	0.4815–1.3773	0.0006
	MatISI	3.62	1.48	256	0.3096	0.3889	0.0000–1.0000	0.2170
	Insulinogenic index 30	2.05	1.71	261	0.8207	0.2425	0.3454–1.2960	0.0034
	Disposition index 30	6.32	4.26	256	0.6200	0.2337	0.1619–1.0781	0.0066
	Insulinogenic index 120	3.12	11.36	273	0.2454	0.2344	0.0000–0.7048	0.1452
	Disposition index 120	9.82	44.10	258	0.4393	0.2273	0.0000–0.8848	0.0276
	ISI_0,120_	85.46	30.56	267	0.0000 #	0.0000	---	0.5000
	Bogalusa insulin sensitivity index	548.53	680.15	272	0.6527	0.2728	0.1180–1.0000	0.0135
	Impaired glucose tolerance ^†^	0.12	0.32	277	0.3704	1.4154	0.0000–1.0000	0.2142

* Sample sizes vary from variable to variable based on the availability of data; Block FFQ data were available for 508 individuals, fasting clinical data for 630 individuals, and OGTT data for 418 individuals. ** Confidence intervals have been truncated to the theoretical bounds of heritability. # Standard errors for heritability estimates could not be estimated since the upper or lower bound for parameters space was reached. ^†^ Sign of the regression coefficients reported by SOLAR has been inverted for ease of interpretation. Also, the 95% confidence interval has been truncated to theoretical bounds of heritability.

## Data Availability

The data reported in this study are available on request from the Corresponding author due to privacy and ethical issues.

## Supplementary Materials

The following supporting information can be downloaded at https://www.mdpi.com/article/10.3390/nu18142361/s1, Supplementary Table S1. Sensitivity analyses using daily calorie intake as a covariate in addition to those mentioned in Figure 1; Supplementary Table S2. Comparison of key characteristics of study participants on whom OGTT data was available with all participants included in the study.

## Abbreviations

The following abbreviations are used in this manuscript:

AUC_Glucose	Area under glucose curve
AUC_Insulin	Area under insulin curve
CMRT	Cardiometabolic risk trait
CO	Childhood obesity
FFQ	Food Frequency Questionnaire
HDL	High density lipoprotein
HOMA_β	Homeostatic model assessment—β cell
HOMA_IR	Homeostatic model assessment—insulin resistance
HOMA_s	Homeostatic model assessment—insulin sensitivity
IFG	Impaired fasting glucose
IGT	Impaired glucose tolerance
MatISI	Matsuda insulin sensitivity index
MET	Metabolic equivalents
OGTT	Oral glucose tolerance test
QUICKI	Quantitative insulin-sensitivity check index
SAFARI	San Antonio Family Assessment of Metabolic Risk Indicators in Youth
UPFs	Ultra-processed foods
