# Supplementary material for: Association of Ultra-Processed Food Intake with Insulin Secretion and Sensitivity in Mexican American Children: Results from the SAFARI Study
Source: Nutrients. 2026 Jul 18;18(14):2361. doi: 10.3390/nu18142361 (PMC13414596; doi:10.3390/nu18142361)
Supplement: Supplementary file 1 [file nutrients-18-02361-s001.zip › nutrients-4355874-supplementary.pdf]

SUPPLEMENTARY MATERIALS FOR

Association of Ultra-Processed Food Intake with Insulin Secretion and Sensitivity in Mexican American Children: Results from The SAFARI Study

Supplementary Table S1. Sensitivity analyses using daily calorie intake as a covariate in addition to those mentioned in Figure 1.

Trait Group	Trait*	Association statistics			
		β	SE(β)	95% CI	p
Fasting	Fasting glucose (mg/dL)	0.1957	0.0820	0.0349-0.3565	0.0170
	Fasting insulin (IU/ml)	0.0272	0.0303	-0.0322-0.0866	0.3689
	QUICKI	-0.0534	0.0471	-0.1457-0.0389	0.2565
	HOMA_β (%)	-0.0703	0.0559	-0.1798-0.0392	0.2082
	HOMA_s (%)	-0.0261	0.0295	-0.0839-0.0317	0.3755
	HOMA_IR	0.0394	0.0391	-0.0373-0.1161	0.3139
	TyG index	0.0313	0.0137	0.0045-0.0582	0.0221
	C_Peptide (ng/ml)	-0.1153	0.0703	-0.2530-0.0224	0.1008
	Impaired fasting glucose†	0.4644	0.2103	0.0522-0.8766	0.0272
OGTT	AUC_Glucose (mg*min/dL) x100	0.0032	0.0046	-0.0059-0.0123	0.4889
	AUC_Insulin (IU*min/ml) x1000	-0.0427	0.0441	-0.1291-0.0438	0.3333
	MatISI	-0.0162	0.0208	-0.0570-0.0246	0.4353
	Insulinogenic Index 30	0.2564	0.0983	0.0638-0.4490	0.0091
	Disposition Index 30	0.2944	0.1118	0.0752-0.5135	0.0085
	Insulinogenic Index 120	0.0146	0.0193	-0.0232-0.0523	0.4502
	Disposition Index 120	0.0104	0.0143	-0.0177-0.0385	0.4672
	$ISI_{0,120}$	0.0927	0.0692	-0.0430-0.2284	0.1805
	Bogalusa insulin sensitivity index	-0.0616	0.0568	-0.1730-0.0498	0.2783
	Impaired glucose tolerance†	0.0999	0.1012	-0.0985-0.2983	0.3236

Supplementary Table S2. Comparison of key characteristics of study participants on whom OGTT data was available with all participants included in the study.

	N	Mean (SD)/ N(%)*	N	Mean (SD) / N(%)
Age (y)	508	11.40 (3.48)	303	12.62 (3.24)
BMI (Kg/m^2)	507	22.30 (6.16)	303	23.68 (6.20)
Waist circumference (cm)	504	75.70 (17.7)	302	80.0 (17.1)
Systolic blood pressure (mmHg)	508	103.99 (10.02)	303	105.44 (81.5)
Diastolic blood pressure (mmHg)	508	62.76 (7.16)	303	63.37 (6.96)
Total serum cholesterol (mg/dl)	474	147.73 (26.85)	299	143.30 (24.76)
Serum high density lipoprotein cholesterol (mg/dl)	472	45.39 (10.92)	298	43.73 (10.11)
Serum triglycerides (mg/dl)	469	73.83 (35.72)	296	77.00 (37.06)
Physical activity (MET score)	477	2.05 (1.30)	296	1.99 (1.23)
UPF Score	508	120.58 (33.60)	303	121.82 (35.08)
Fasting glucose (mg/dL)	480	89.74 (7.08)	303	89.62 (6.58)
Fasting insulin (IU/ml)	472	12.81 (6.99)	299	13.14 (7.22)
QUICKI	469	0.34 (0.03)	299	0.33 (0.03)
HOMA_β (%)	475	147.82 (60.61)	302	151.26 (62.52)
HOMA_s (%)	475	69.98 (45.48)	302	68.42 (46.39)
HOMA_IR	471	1.86 (0.98)	299	1.91 (1.00)
TyG index	464	8.00 (0.47)	296	8.04 (0.46)
Serum C_Peptide (ng/ml)	403	0.95 (0.76)	247	0.99 (0.79)
